# Supplementary material for: The Analysis of Trends in Survival for Patients with Melanoma Brain Metastases with Introduction of Novel Therapeutic Options before the Era of Combined Immunotherapy—Multicenter Italian–Polish Report
Source: Cancers (Basel). 2022 Nov 23;14(23):5763. doi: 10.3390/cancers14235763 (PMC9737166; doi:10.3390/cancers14235763)
Supplement: Supplementary file 1 [file cancers-14-05763-s001.zip › cancers-1973042-Supplementary.pdf]

The analysis of trends in survival for patients with melanoma brain metastases with introduction of novel therapeutic options before the era of combined immunotherapy–multicenter Italian-Polish report.

Supplementary Materials

Joanna Placzke[a#], Pawel Teterycz[af#], Pietro Quaglino[c], Bozena Cybulska-Stopa[d], Marco Tucci[e], Marco Rubatto[c], Tomasz Skora[d], Valeria Interno[h], Magdalena Rosinska[f], Aneta Borkowska[a], Anna Szumera-Cieckiewicz[g], Mario Mandala[b✦], Piotr Rutkowski[a✦]

Figure S1. MICE imputation diagnostics. Records are color-codded into observed (blue) and imputed (red) data.

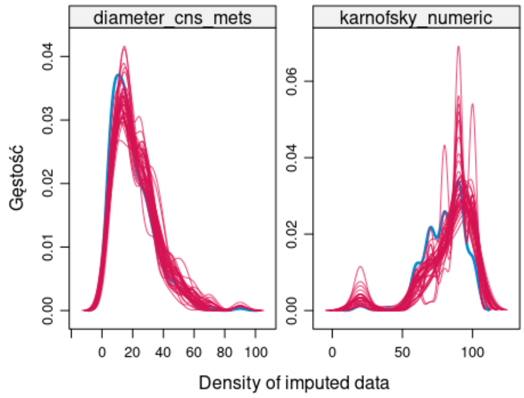

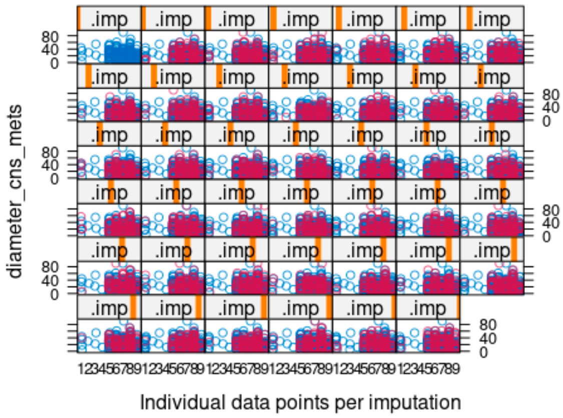

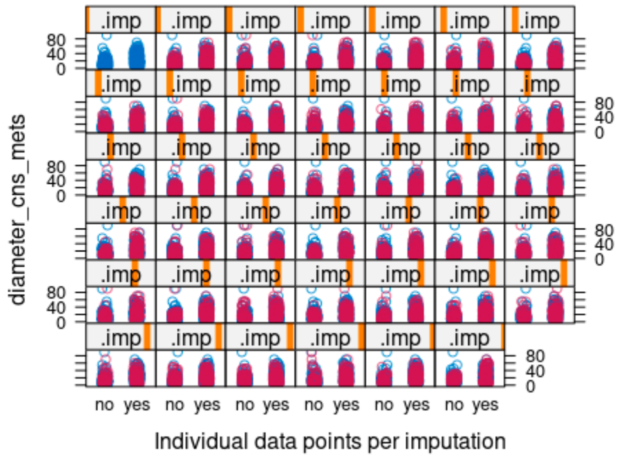

Table S1. Patients' breakdown by granted mol-GPA score.

	level	Overall, number (%)	Missing
**Treatment part**			
	level	Overall, number (%)	Missing
		402	
gpa_mol score	0	5 (1.2)	0.0
	0.5	24 (6.0)	
	1	52 (12.9)	
	1.5	55 (13.7)	
	2	110 (27.4)	
	2.5	68 (16.9)	
	3	46 (11.4)	
	3.5	28 (7.0)	
	4	14 (3.5)	
**Prognostic part**			
	level	Overall, number (%)	Missing
		487	
gpa_mol score	0	5 (1.2)	12.9
	0.5	24 (5.7)	
	1	56 (13.2)	
	1.5	58 (13.7)	
	2	114 (26.9)	
	2.5	75 (17.7)	
	3	47 (11.1)	
	3.5	31 (7.3)	
	4	14 (3.3)	

Table S2. Median overall survival Depending on mol-GPA.

strata	Median OS	lower 95% CI	upper 95% CI
mol GPA0.0-1.0 before 2017	2.7	1.9	5.2
mol GPA0.0-1.0 since 2017	4.6	2.8	8.5
mol GPA1.5-2.0 before 2017	5.1	4.2	6.3
mol GPA1.5-2.0 since 2017	6.3	4.8	9.3
mol GPA2.5-3.0 before 2017	9.3	6.9	12.3
mol GPA2.5-3.0 since 2017	8.5	6.7	12.1
mol GPA3.5-4.0 before 2017	15.6	9.0	35.0
mol GPA3.5-4.0 since 2017	21.1	13.8	NA

Table S3. The complete comparison of treatment selection regarding treatment modalities before or after 30 days from MBM diagnosis.

Dependent:		Number of pts (%)	HR (univariable)
Surgery within 30 days	no_surgery	343 (85.3)	-
	surgery_in_30days	40 (10.0)	0.61 (0.43-0.88, p=0.009)
	surgery_outside_30d	19 (4.7)	0.53 (0.31-0.91, p=0.022)
Rth within 30 days	no_rth	93 (23.1)	-
	outside_30d	1 (0.2)	0.66 (0.09-4.77, p=0.683)
	srs_in_30days	37 (9.2)	0.45 (0.29-0.70, p<0.001)
	srs_outside_30d	48 (11.9)	0.36 (0.24-0.55, p<0.001)
	wbrt_in_30days	148 (36.8)	1.42 (1.08-1.87, p=0.012)
	wbrt_outside_30d	75 (18.7)	0.97 (0.70-1.34, p=0.856)
Systemic treatment within 30 days	no_systemic	94 (23.4)	-
	systemic_in_30days	145 (36.1)	0.34 (0.26-0.45, p<0.001)
	systemic_outside_30d	163 (40.5)	0.26 (0.20-0.34, p<0.001)
Any treatment within 30 days	no_trt	109 (27.1)	-
	rth	115 (28.6)	1.46 (1.10-1.92, p=0.008)
	rth_systemic	67 (16.7)	1.11 (0.80-1.54, p=0.547)
	surgery	30 (7.5)	0.71 (0.45-1.10, p=0.125)
	systemic	71 (17.7)	0.99 (0.71-1.37, p=0.941)
	Other	10 (2.5)	0.80 (0.37-1.72, p=0.565)

Table S4. The complete comparison of treatment selection regarding the clinical features.

	no_trt	rth	rth_systemic	surgery	systemic	p
n	109	115	67	30	71	
age (median [IQR])	56.00 [49.00, 66.00]	57.00 [46.50, 67.00]	55.00 [43.00, 61.50]	56.50 [43.25, 66.50]	55.00 [43.00, 68.50]	0.555
sex = male (%)	65 (59.6)	64 (55.7)	37 (55.2)	19 (63.3)	44 (62.0)	0.848
braf = wt (%)	42 (38.5)	50 (43.5)	23 (34.3)	9 (30.0)	21 (29.6)	0.320
n_cns_mets (%)						<0.001
1	28 (25.7)	20 (17.4)	13 (19.4)	16 (53.3)	13 (18.3)	
2	15 (13.8)	7 (6.1)	5 (7.5)	7 (23.3)	14 (19.7)	
3	10 (9.2)	12 (10.4)	4 (6.0)	3 (10.0)	10 (14.1)	
4	4 (3.7)	3 (2.6)	1 (1.5)	0 (0.0)	3 (4.2)	
5+	52 (47.7)	73 (63.5)	44 (65.7)	4 (13.3)	31 (43.7)	
diameter_cns_mets (median [IQR])	15.00 [8.00, 25.00]	18.00 [12.00, 27.75]	16.00 [10.50, 24.50]	33.00 [24.50, 43.50]	15.00 [9.25, 20.50]	<0.001
Previous treatment (%)						0.011
0	71 (65.1)	60 (52.2)	43 (64.2)	23 (76.7)	50 (70.4)	
1	21 (19.3)	34 (29.6)	22 (32.8)	5 (16.7)	15 (21.1)	
2+	17 (15.6)	21 (18.3)	2 (3.0)	2 (6.7)	6 (8.5)	

Table S5. Univariable Cox models for overall survival depending on systemic treatment selection.

Dependent:		Number of pts (%)	HR (univariable)
had_brafmek	no	372 (92.5)	-
	yes	30 (7.5)	1.57 (1.06-2.30, p=0.023)
had_chth	no	368 (91.5)	-
	yes	34 (8.5)	1.60 (1.11-2.31, p=0.011)
had_braf	no	372 (92.5)	-
	yes	30 (7.5)	1.41 (0.96-2.06, p=0.079)
had_ipi	no	377 (93.8)	-
	yes	25 (6.2)	1.38 (0.90-2.10, p=0.139)
had_pd1	no	377 (93.8)	-
	yes	25 (6.2)	1.03 (0.67-1.59, p=0.885)
